# Detection of an unusual G8P[8] rotavirus in a Rotarix-vaccinated child with acute gastroenteritis using Nanopore MinION sequencing: A case report

**DOI:** 10.1097/MD.0000000000022641

**Published:** 2020-10-02

**Authors:** Jong-Hwa Kim, Dae Yong Yi, Inseok Lim, Alan C. Ward, Wonyong Kim

**Affiliations:** a Department of Microbiology; b Department of Pediatrics, Chung-Ang University College of Medicine, Seoul, South Korea; c School of Biology, Newcastle University, Newcastle upon Tyne, UK.

**Keywords:** acute gastroenteritis, diagnosis, G8P[8], MinION sequencing, rotavirus

## Abstract

**Rationale:**

Rotavirus is routinely diagnosed by the detection of antigens or the viral genome. However, these tests have limitations, in that they do not detect all rotavirus strains.

**Patient concerns:**

We present a case of a 27-month-old girl who was hospitalized for 4 days with severe gastroenteritis, including high fever, vomiting, diarrhea, mild dehydration, and periumbilical pain. Notably, the patient previously received the Rotarix vaccine.

**Diagnoses:**

The laboratory tests were negative for rotavirus, astrovirus, adenovirus, and norovirus as well as common diarrhea-causing bacteria. Human-bovine recombinant rotavirus was detected by MinION sequencing.

**Interventions:**

To investigate the cause agents from the unexplained severe gastroenteritis infant, the stool sample was prepared by random amplification for Nanopore MinION sequencing.

**Outcomes:**

Treatment through the administration of ORS solution and galtase powder with probiotics was successful after the diagnosis of unusual rotavirus infection.

**Lessons:**

This case report is the first detection of an unusual human-bovine recombinant rotavirus in an idiopathic gastroenteritis using Nanopore MinION sequencing.

## 1 Introduction

Rotaviruses are the major cause of diarrhea-associated hospitalization and death among children less than 5 years of age. Nearly 200,000 children die from rotavirus diarrhea each year.^[1]^ Accurate and rapid diagnosis of rotavirus infection is important to for the determination of appropriate treatment control of the spread of infection. Laboratory diagnosis of rotavirus infection is usually performed using diarrheal stool samples from symptomatic patients using enzyme immunoassays and reverse transcription polymerase chain reaction.^[2]^ However, these methods are not always able to detect all rotavirus strains and have difficulty in distinguishing between wild-type and vaccine strains, a distinction which is crucial for clinical samples.[^3^,^4^] Furthermore, new rotaviruses (human and animal recombinant strains) have emerged, which cannot be detected by conventional methods.^[5]^ These concerns have been raised about whether the rotavirus detection might fail to detect all rotavirus cases and thus lead to misdiagnosis of some cases. Nanopore MinION sequencing is a third-generation single-molecule sequencing technology that allows for rapid genome analysis of bacterial and viral isolates.^[6]^ Recently, Nanopore MinION sequencing has been used to detect viruses, such as Ebola virus,^[7]^ and a new recombinant hepatitis B virus.^[8]^ However, the detection of rotavirus has not been reported. Here, we report the first application of Nanopore MinION sequencing for the diagnosis of rotavirus infection via the detection of an unusual human-bovine recombinant rotavirus causing a gastroenteritis for which the causative organism could not be detected by the usual diagnostic methods in a Rotarix-vaccinated child.

## 2 Case presentation

A 27-month-old girl patient was hospitalized for 4 days with severe gastroenteritis in January 2018 to the Department of Pediatrics at Chung-Ang University Hospital (Seoul, South Korea). The patient had severe signs and symptoms, including vomiting (twice per day), diarrhea (twice per day), high fever (38.5°C), mild dehydration, and periumbilical pain. At the time, the patient had a hemoglobin level of 12.2 g/dL, a white blood cell count of 8280 cells/μL, an absolute neutrophil count of 5067 cells/μL, a serum C-reactive protein level of 19.6 mg/dL, an erythrocyte sedimentation rate of 21 mm/h, a serum aspartate aminotransferase level of 50 IU/L, and a serum alanine aminotransferase level of 20 IU/L. Of note, the patient was given the Rotarix vaccine in 2 doses at the ages of 2 and 4 months.

Stool samples were collected according to a protocol (#1710-009-303) approved by the Human Subjects Institutional Review Board of Chung-Ang University Hospital. Written informed consent was obtained from the parents of the infant enrolled in the study for data provision and publication of this case report.

The stool sample was pre-diagnosed as negative for rotavirus using an RIDASCREEN Rotavirus kit (R-Biopharm AG, Germany) and was negative for astrovirus, rotavirus, enteric adenovirus, and norovirus according to the Seeplex Diarrhea-V ACE Detection Assay (Seegene, Korea), from a stool specimen taken at a diagnostic laboratory in the Department of Laboratory Medicine of Chung-Ang University Hospital, Seoul, Korea. Moreover, stool culture confirmed that there was no growth of common diarrhea-causing bacteria, such as diarrheogenic *Escherichia coli*, *Salmonella*, *Shigella*, and *Campylobacter*.

**Figure 1:**
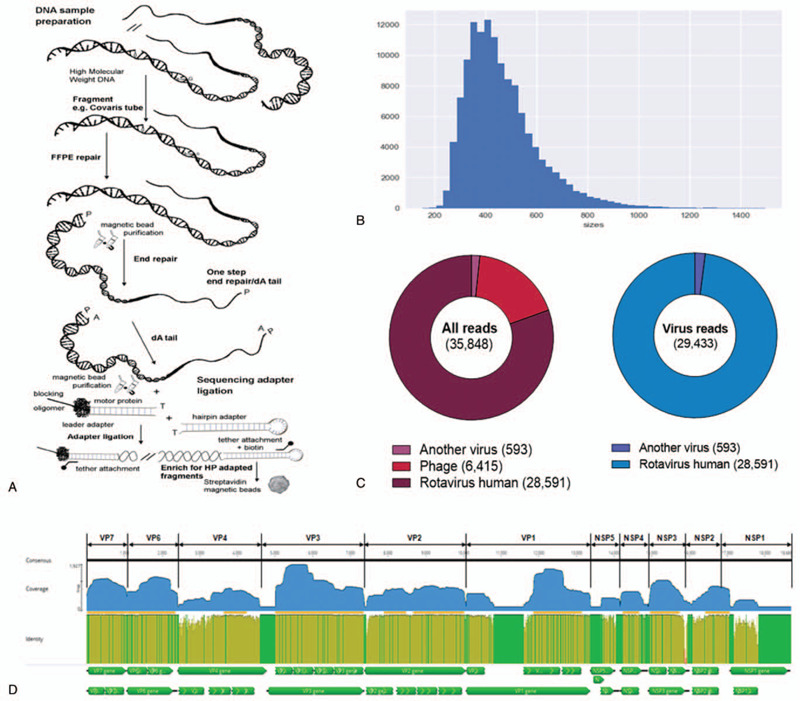
Detection of rotavirus by MinION sequencing. The figures indicated (A) example protocol for SQK-MAP006 (*6*), (B) the histogram of read counts between 200 and 1500 bp in length, (C) taxonomy of total number of reads and taxonomy of virus reads, and (D) an assembly of G8P[8] rotavirus 11 gene segments using Geneious R10 for mapping.

To confirm the unexplained severe gastroenteritis patient, the stool specimen was immediately transported to the Department of Microbiology, Chung-Ang University College of Medicine. The stool sample was suspended in 10% phosphate buffered saline and centrifuged at 12,000 *g* for 15 minutes, and the supernatant was collected. Viral RNA was extracted from the supernatant after filtration through a 0.2 μm sterile syringe filter (Corning, Corning, NY) using the QIAamp Viral RNA Mini Kit (Qiagen, Hilden, Germany). The viral RNA was used to construct a library for Nanopore MinION sequencing [Oxford Nanopore Technologies, Cambridge, UK (Fig. 1A)]. FASTQ files, generated by MinION sequencing, were used to assess sequence quality with poretools software (v. 0.6.0). The library was constructed, and low-quality sequences, which included adapter sequences and short reads (< 20 bp), were trimmed using Geneious R10 (v.10.0.9). Phylogenetic analysis and amino acid identification were performed using MEGA X software.

A total of 144,406 sequence reads were obtained with a mean length of 514.9 ± 58.9 bp after trimming adapter sequences and filtering based on quality and read length (Fig. 1B). Of a total of 35,848 reads, 29,433 reads were aligned to virus sequences from the NCBI database and were matched to the human rotavirus genome (97.7% of virus reads) (Fig. 1C). The aligned sequences covered 11 segments of G8P[8] group A rotavirus and indicated a high genome coverage (%) with >95.0% of pairwise identity (Fig. 1D). The mapped reference sequence/total length of VP6, VP7, NSP2, and NSP3 showed a high coverage of 100.0% (Table 1). In addition, VP2, VP4, and NSP4 showed a coverage >95.0%. VP3 showed a coverage of 91.4% per mapped reference sequence. On the other hand, VP1, NSP5, and NSP1 showed a coverage of only 76.1%, 59.1%, and 40.0%, respectively. To classify the detected rotavirus strain (designated as CAU17L-79) within group A rotavirus, representative sequences from each segment were extracted, and a phylogenetic tree was constructed with each reference sequence. All 11 segments of the CAU17L-79 strain were aligned and mapped to human G8P[8] rotavirus strains in GenBank: NP-130/2014 and KKL-17/2013 from Thailand, RVN1149/2014 from Vietnam, and SO1162/2017 from Japan. However, the VP7 gene was from bovine G8P[14] rotavirus (Cow 68/2007 and Cow-UP/2010 from India). The CAU17L-79 strain was found to possess the G8-P[8]-I2-R2-C2-M2-A2-N2-T2-E2-H2 genotype constellation for the VP7-VP4-VP6-VP1-VP2-VP3-NSP1-NSP2-NSP3-NSP4-NSP5/6 genes, respectively (GenBank accession no. MN058730-MN058740). Therefore, CAU17L-79 is a strain responsible for multiple reassortment events between the DS-1–like P[8] strains and bovine strains in Asia (Fig. 2 ). After the diagnosis of rotavirus infection by MinION sequencing, the patient was successfully treated by rehydration with low-osmolarity ORS solution and galtase powder with probiotics after 3 days.

**Table 1:**
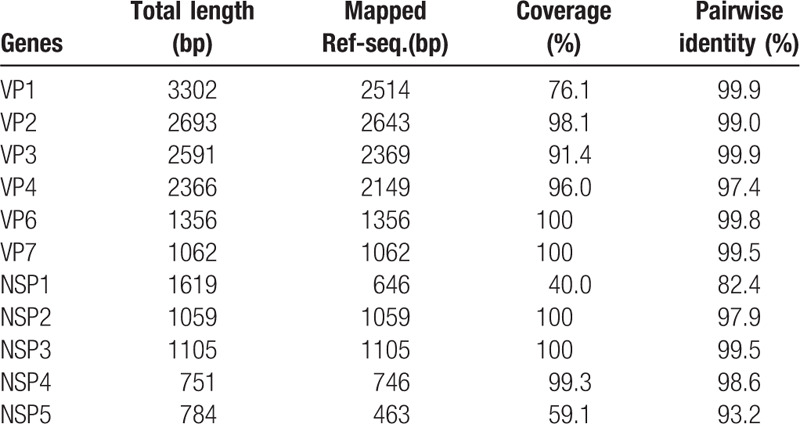
Sequence coverage of G8P[8] rotavirus 11 gene segments using MinION sequencing.

**Figure 2:**
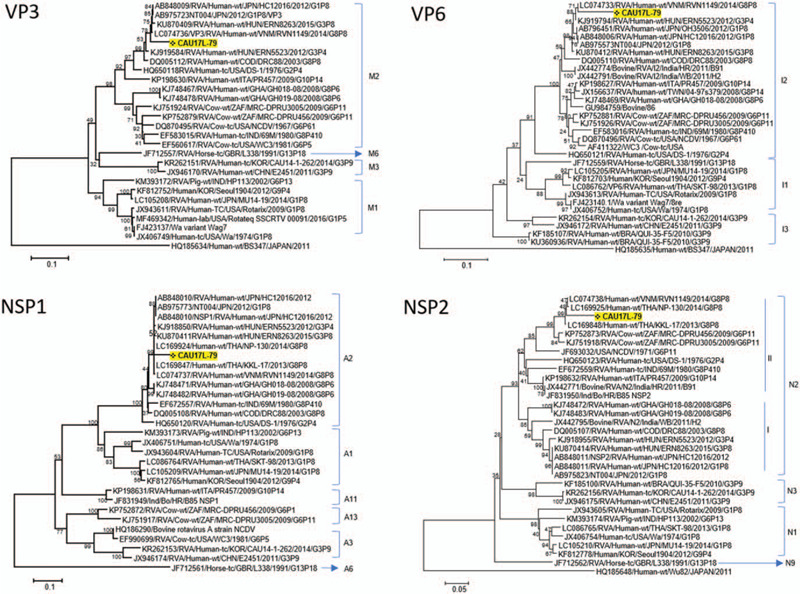
Phylogenetic tree of G8P[8] rotavirus based on nucleotide sequences obtained from MinION sequencing. The phylogenetic tree was constructed from 11 rotavirus gene sequences (VP7, VP4, VP1, VP2, VP3, VP6, NSP1, NSP2, NSP3, NSP4, and NSP5).

**Figure 2 (Continued):**
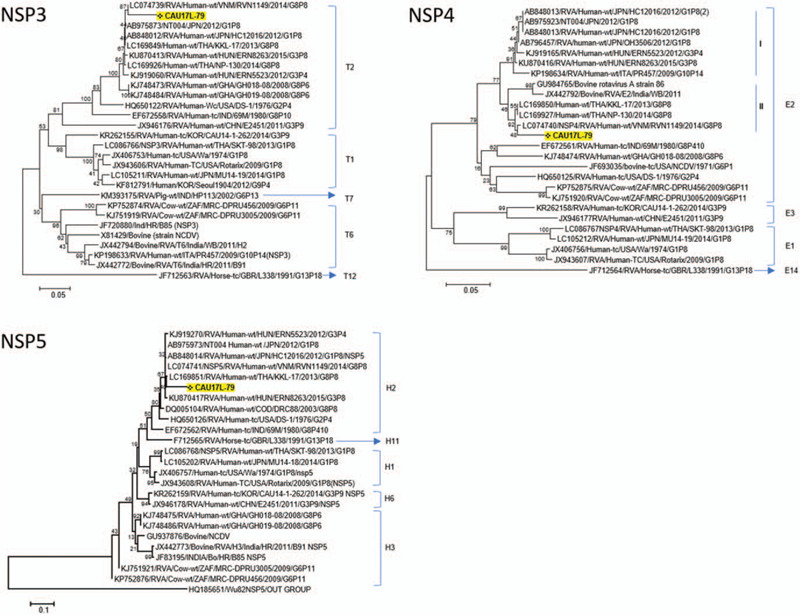
Phylogenetic tree of G8P[8] rotavirus based on nucleotide sequences obtained from MinION sequencing. The phylogenetic tree was constructed from 11 rotavirus gene sequences (VP7, VP4, VP1, VP2, VP3, VP6, NSP1, NSP2, NSP3, NSP4, and NSP5).

## 3 Discussion

In 2006, 2 licensed vaccines, Rotarix, a human monovalent G1P[8] vaccine, (GlaxoSmithKline, Belgium) and RotaTeq, a pentavalent human-bovine reassortant vaccine (Merck & Co.) were used in more than 100 countries.^[9]^ Both Rotarix and RotaTeq have been found to be effective against common circulating strains such as the G1, G3, G4, and G9 strains of P[8].^[10]^ However, these vaccines are not sufficient for protection against all rotavirus G and P serotypes, which are not included in the vaccine formulation.[^11^,^12^,^13^]

It is of note that a G8P[8] rotavirus patient in this study was given Rotarix according to a standard schedule. The current rotavirus vaccines commercially available do not include this genotype. Considering that G8P[8] rotaviruses appear to be emerging in recent years,^[14]^ concerns have been raised about failures in preventing and diagnosing rotavirus infections, particularly in the post-vaccine era.

To overcome these limitations, large-scale complex genome sequencing for obtaining sequences for all 11 rotavirus RNA segments is needed. Nanopore MinION sequencing is an emerging third-generation sequencing technology, which offers rapid microbial identification. It has 2 advantages: the ability to perform real-time sequence analysis enable metagenomic species identification; and the capacity to obtain longer reads, offering simplified and less ambiguous genome assembly.^[6]^

In this study, although the sample was pre-diagnosed as negative for various enteric microbes in the hospital, an unusual G8P[8] rotavirus (possibly acquired via bovine-to-human interspecies transmission) was detected with the identification of 11 gene segments for <3 hours. The findings indicate that Nanopore MinION sequencing may be used as a rapid diagnostic tool in cases of idiopathic severe gastroenteritis and in epidemiological studies to improve our understanding of the origin, genetic recombination, and burden of disease simultaneously. More studies using this method are needed to determine the efficacy and effectiveness of current vaccines based on global viral surveillance.

## Acknowledgment

We would like to thank Miss Jihye Baek for support with data analysis.

## Author contributions

**Collection of clinical data and stool samples:** Dae Yong Yi, Inseok Lim.

**Conceptualization and design:** Wonyong Kim, Alan C. Ward.

**Data curation:** Dae Yong Yi, Inseok Lim, Alan C. Ward, Wonyong Kim.

**Formal analysis:** Alan C. Ward, Wonyong Kim.

**Funding acquisition:** Wonyong Kim.

**Investigation:** Alan C. Ward, Wonyong Kim.

**Laboratory work:** Jong-Hwa Kim.

**Methodology:** Jong-Hwa Kim.

**Project administration:** Wonyong Kim.

**Resources:** Dae Yong Yi, Inseok Lim.

**Software:** Alan C. Ward, Wonyong Kim.

**Statistical analysis:** Wonyong Kim, Alan C. Ward.

**Supervision:** Inseok Lim, Wonyong Kim.

**Validation:** Dae Yong Yi, Alan C. Ward, Wonyong Kim.

**Writing – original & draft:** Wonyong Kim, Jong-Hwa Kim.

**Writing – review & editing:** Inseok Lim, Alan C. Ward.

## References

[1] Hasso-Agopsowicz M Ladva CN Lopman B Global review of the age distribution of rotavirus disease in children aged < 5 years before the introduction of rotavirus vaccination. Clin Infect Dis 2019;69:1071–8. 30689799 10.1093/cid/ciz060 PMC6736387

[2] World Health Organization. Manual of Rotavirus Detection and Characterization Methods. No. WHO/IVB/08.17. Geneva: World Health Organization; 2009.

[3] Parashar UD Nelson EA Kang G Diagnosis, management, and prevention of rotavirus gastroenteritis in children. BMJ 2013;347:f7204. 24379214 10.1136/bmj.f7204 PMC5776699

[4] Jeong S Than VT Lim I Differentiation of RotaTeq® vaccine strains from wild-type strains using NSP3 gene in reverse transcription polymerase chain reaction assay. J Virol Methods 2016;237:72–8. 27590978 10.1016/j.jviromet.2016.08.022

[5] Thanh HD Tran VT Lim I Emergence of human G2P[4] rotaviruses in the post-vaccination era in South Korea: footprints of multiple interspecies re-assortment events. Sci Rep 2018;8:6011. 29662148 10.1038/s41598-018-24511-y PMC5902508

[6] Ward AC Kim W MinION^TM^: new, long read, portable nucleic acid sequencing device. J Bacteriol Virol 2015;45:285–303.

[7] Quick J Loman NJ Duraffour S Real-time, portable genome sequencing for Ebola surveillance. Nature 2016;530:228–32. 26840485 10.1038/nature16996 PMC4817224

[8] Sauvage V Boizeau L Candotti D Early MinION^TM^ nanopore single-molecule sequencing technology enables the characterization of hepatitis B virus genetic complexity in clinical samples. PLoS One 2018;13:e0194366. 29566006 10.1371/journal.pone.0194366 PMC5864009

[9] Kirkwood CD Genetic and antigenic diversity of human rotaviruses: potential impact on vaccination programs. J Infect Dis 2010;202:S43–8. 20684716 10.1086/653548

[10] Soares-Weiser K Maclehose H Bergman H Vaccines for preventing rotavirus diarrhoea: vaccines in use. Cochrane Database Syst Rev 2012;14:CD008521. 10.1002/14651858.CD008521.pub2 22336845

[11] Trojnar E Sachsenroder J Twardziok S Identification of an avian group A rotavirus containing a novel VP4 gene with a close relationship to those of mammalian rotaviruses. J Gen Virol 2013;94:136–42. 23052396 10.1099/vir.0.047381-0

[12] Tissera MS Cowley D Bogdanovic-Sakran N Options for improving effectiveness of rotavirus vaccines in developing countries. Hum Vaccin Immunother 2017;13:921–7. 27835052 10.1080/21645515.2016.1252493 PMC5404363

[13] Block SL Vesikari T Goveia MG Efficacy, immunogenicity, and safety of a pentavalent human-bovine (WC3) reassortant rotavirus vaccine at the end of shelf life. Pediatrics 2007;119:11–8. 17200266 10.1542/peds.2006-2058

[14] Hoque SA Kobayashi M Takanashi S Role of rotavirus vaccination on an emerging G8P[8] rotavirus strain causing an outbreak in central Japan. Vaccine 2018;3:43–9. 10.1016/j.vaccine.2017.11.056 29183732

